# Induction of Metallothionein Expression After Exposure to Conventional Cigarette Smoke but Not Electronic Cigarette (ECIG)-Generated Aerosol in *Caenorhabditis elegans*

**DOI:** 10.3389/fphys.2018.00426

**Published:** 2018-04-23

**Authors:** Eric Cobb, Julie Hall, Dominic L. Palazzolo

**Affiliations:** ^1^School of Mathematics and Sciences, Lincoln Memorial University, Harrogate, TN, United States; ^2^DeBusk College of Osteopathic Medicine, Lincoln Memorial University, Harrogate, TN, United States; ^3^Department of Biology, School of Mathematics and Sciences, Lincoln Memorial University, Harrogate, TN, United States; ^4^Department of Physiology, DeBusk College of Osteopathic Medicine, Lincoln Memorial University, Harrogate, TN, United States

**Keywords:** ECIG, E-liquid, smoking, *C. elegans*, metallothionein, stress-induced sleep

## Abstract

**Aim:** With the invention of electronic cigarettes (ECIG), many questions have been raised regarding their safety as an alternative to smoking conventional cigarettes. Conventional cigarette smoke contains a variety of toxicants including heavy metals. However, ECIG-generated aerosol contains only trace amounts of metals, adding to the argument for it being a safer alternative. In response to heavy metal exposure, metallothioneins are induced in cells to help store the metal, detoxify the body, and are also known responders to oxidative stress. In an attempt to add to the evaluation of the safety of ECIGs, metallothionein expression was quantified using the nematode *Caenorhabditis elegans* as an assessment of stress induced cellular damage caused by exposure.

**Methods:** Adult nematodes were exposed to either ECIG aerosol or conventional cigarette smoke at doses of 15, 30, and 45 puffs, the equivalent of one, two, and three cigarettes, respectively. Movement, survival, and stress-induced sleep were assessed for up to 24 h after exposure. Relative expression levels for *mtl-1* and *mtl-2, C. elegans* metallothionein genes, were analyzed after 1, 5, and 24 h post exposure using quantitative RT-PCR.

**Results:** Nematodes exposed to conventional cigarette smoke underwent stress-induced sleep in a dose dependent manner with animals recovering to values within the range of air control after 5 h post exposure. Those exposed to ECIG aerosol did not undergo stress-induced sleep and were indistinguishable from controls. The expression of *mtl-1* increased in a dose and time dependent manner in *C. elegans* exposed to conventional cigarette smoke, with a maximum expression observed at 5 h post exposure of 45 puffs. No induction of *mtl-2* was observed in any animals. Additionally, ECIG aerosol did not induce expression of *mtl-1* and *mtl-2* at levels different than those of untreated.

**Conclusion:** ECIG aerosol failed to induce a stress response in *C. elegans*. In contrast, conventional cigarette smoke induced the production of *mtl-1* in a manner that correlates with the induction of stress-induced sleep suggesting a stress response to damage. The lack of cellular stress response to ECIG aerosol suggests it may be a safer alternative to conventional cigarettes.

## Introduction

Cigarette smoking is responsible for hundreds of thousands of deaths per year and increases the risk of cardiovascular disease, stroke, respiratory disease, and cancer. Smoke produced by conventional cigarettes contains thousands of toxic and carcinogenic chemicals including, but not limited to: benzene, cyanide, carbon monoxide, nitrosamines, heavy metals, and even radioactive elements (Talhout et al., 2011). Electronic cigarettes (ECIGs) are becoming an increasingly more popular alternative due to the public perception that they are ‘healthier’ than conventional cigarettes. A significant increase in both awareness and usage of ECIGs among smokers and non-smokers was seen between 2010 and 2015 (King et al., 2015a,b).

There are currently three generations of ECIG devices; although there are differences in the core assemblies and abilities, they all operate by the same underlying mechanism. A voltage source produces an electric current that heats an atomizer consisting of a resistance coil surrounding a wick. The atomizer heats ECIG liquid (E-liquid) to its vaporization point producing an aerosol for inhalation (Williams et al., 2013, 2017; Palazzolo et al., 2017a). E-liquids generally contain a humectant such as glycerol or propylene glycol, distilled water, nicotine, and flavorings. Under normal vaping conditions (Bates and Farsalinos, 2015; Holliday et al., 2016), there is little evidence to support adverse effects in response to ECIG-generated aerosol, especially when compared to conventional cigarette smoke. However, trace amounts of metals in ECIG aerosol have been reported at levels significantly lower than those found in conventional cigarette smoke (Williams et al., 2013, 2017; Czoli et al., 2015; Palazzolo et al., 2017a). These metals are hypothesized to originate from the metal components of the atomizer and include: aluminum (Al), arsenic (As), cadmium (Cd), copper (Cu), iron (Fe), manganese (Mn), nickel (Ni), lead (Pb), and zinc (Zn) (Williams et al., 2013, 2017; Palazzolo et al., 2017a). While Aherrera et al. (2017) very recently reported positive associations of Ni and Chromium (Cr) aerosol concentrations with corresponding Ni and Cr biomarker levels in urine and saliva, their results (along with the aforementioned studies) suggest absorption of these metals from cigarette smoke would present a greater physiological problem.

Trace metals such as Cd, Zn, and Cu are known to induce transcription of and bind to metallothioneins (MT). MTs are a family of highly conserved small, cysteine rich, metal binding proteins. They transiently bind monovalent and bivalent essential trace metals such as Zn, Cu, and Mn as well as non-essential metals such as Cd and mercury (Hg) (Aschner and Martinez-Finley, 2011). They are hypothesized to function in homeostasis and sequestration of essential trace metals, detoxification of non-essential metals, and protection against oxidative damage (Freedman et al., 1993; Vašák, 2005; Aschner and Martinez-Finley, 2011; Isani and Carpenè, 2014). Specifically, increased concentrations of heavy metals, glucocorticoids, cytokines, and reactive oxygen species (ROS), such as hydrogen peroxide, have been reported to up-regulate their transcription (Bauman et al., 1991; Dalton et al., 1996; Vašák, 2005; Zeitoun-Ghandour et al., 2011). Mammals have four different MT isoforms, with MT-1 and MT-2 being the most sensitive to these inducers. Their promoter regions contain both metal and glucocorticoid response elements. Several mechanisms have been proposed for the regulation of stress-induced MT transcription, mainly metal-responsive transcription factor 1 (MTF-1 binding to the metal regulatory element as the key factor (Günther et al., 2012). Various metals bind MTF-1, leading to the increased MT expression needed to restore homeostasis (Vašák, 2005).

Unlike mammals, the nematode *Caenorhabditis elegans* has only two identified MT isoforms, *mtl-1* and *mtl-2.* Additionally, metal response elements are not found in the functional promotor region of *mtl-1* but are present in *mtl-2*. However, the location is not in the minimal promoter region needed for transcription and is thus thought to be non-functional (Freedman et al., 1993; Moilanen et al., 1999). Additionally, no homolog to the MT transcription factor, MTF-1, has been identified in *C. elegans*. The lack of MTF-1 conservation has led to alternative models of MT regulation in *C. elegans*. One model supports that regulation involves enzymes of the insulin signaling pathway and transcription factors ATF-7 and ELT-2 binding of CRE-like and GATA regulator elements. This model suggests that cellular stress in the form of metal toxicity (Cd in particular), positively regulates transcription of MT by promoting the dissociation of ATF-7, followed by subsequent binding of ELT-2 (Moilanen et al., 1999; Shivers et al., 2010; Hall et al., 2017). Although *C. elegans* regulation of MT expression differs from that of higher eukaryotic organisms, *mtl-1* and *mtl-2* are activated under similar conditions and have conserved homologous functions (Thornalley and Vašák, 1985; Slice et al., 1990; Freedman et al., 1993; Zeitoun-Ghandour et al., 2011; Hall et al., 2012).

Using *C. elegans* in a novel approach to study the physiological effects of ECIG-generated aerosol and conventional cigarette smoke, this investigation was designed to gauge the safety level of ECIGs as a ‘harm-reduction’ alternative to conventional cigarettes. The expression of MTs was used as an indirect method to compare the heavy metal levels and/or ROS exposure found in conventional cigarette smoke and ECIG aerosol. Metal toxicity in the smoke and aerosol was assessed using quantitative RT-PCR to measure and compare *mtl-1* and *mtl-2* gene expression levels in *C. elegans*. Additionally, pharyngeal pumping and locomotion were measured as key characteristics of stress-induced sleep (Trojanowski and Raizen, 2016; DeBardeleben et al., 2017). This measurement of health in the nematodes is an attempt to further understand the effects on the whole organism after exposure to smoke and aerosol.

## Materials and Methods

### Strains

The following strains were used: N2 Bristol wild-type. The strain was provided by the *Caenorhabditis* Genetics Center (CGC), which is funded by NIH Office of Research Infrastructure Programs (P40 OD010440). Unless otherwise indicated, all strains were maintained and experiments conducted at 20°C using 60 mm NGM agar plates containing *Escherichia coli* OP50 as a food source (Sulston and Hodgkin, 1988).

Age synchronization of *C. elegans* was accomplished as previously described (Khanna et al., 1997). Briefly, gravid adult nematodes were incubated in alkaline hypochlorite solution (250 μM NaOH, 1% Clorox) to isolate embryos. Embryos were collected by centrifugation and then washed with K medium (32 mM KCl and 51 mM NaCl) (Williams and Dusenbery, 1988). To generate L4 *C. elegans*, embryos were placed on NGM plates with food and allowed to grow for 48 h at 20°C.

### Exposure of Nematodes to Air, Aerosol, or Smoke

Age-synchronized L4 larvae on NGM agar plates containing food were placed into clear cylindrical acrylic exposure chambers uncovered and exposed to 30 puffs air (control), ECIG-generated aerosol, or conventional cigarette smoke. Air, ECIG aerosol, or smoke was pumped into the exposure chambers similar to that previously described (Palazzolo et al., 2017b). Briefly, two Cole-Parmer Master Flex L/S peristaltic pumps (Vernon Hills, IL, United States) were used to simulate puffing on Triple 3 (Kennesaw, GA, United States) eGo style ECIG device or conventional Marlboro (84 mm, full strength) cigarettes. The Triple 3 eGo device, manufactured in China by JOMO Tech (2017), consists of a 650 mAh lithium ion battery (3.7 V, unregulated), a silicon ring at the base of the mouth piece, and a plastic tank (i.e., “clearomizer”) with a 1.6 ml capacity to house the E-liquid. The resistance of the tank’s heating coils varies between 2.2 and 2.6 Ω for an average power output of ≈5.7 W. The ECIG devices vaporized an in-house prepared E-liquid mixture of 50% propylene glycol and 50% vegetable glycerin (i.e., glycerol) containing 20 mg/ml of nicotine, or approximately 2.8 mg nicotine/15 puffs. This concentration is chosen because it has been determined that a concentration of 20 mg/ml nicotine in E-liquid is required to deliver similar amounts of nicotine as conventional cigarettes (Farsalinos et al., 2013). In comparison, a full-strength Marlboro^®^ contains slightly less than 1.0 mg nicotine/cigarette (Calafat et al., 2004). One peristaltic pump (aerosol pump) was used to transport air or mainstream ECIG-generated aerosol through 16 inches of Master Flex L/S 24 Precision Tubing (ID = 6.4 mm) into the exposure chamber. A second peristaltic pump (the smoke pump) was used to transport air or mainstream smoke through an identical setup as the first peristaltic pump. To minimize cross contamination of pump tubing, the aerosol pump was used strictly for aerosol and the smoke pump strictly for smoke. The puffing protocol consisted of up to 45 cycles of a 5 s puff (pump active) followed by a 10 s delay period (pump inactive). Multiple plates were placed in the same exposure chambers for each exposure (aerosol or smoke) and were removed after 15, 30, and 45 puffs. The rubber cap at the end of the chamber was removed to retrieve plates and was replaced within a 5 s interval to minimize the release of aerosol or smoke from the exposure chamber. Control exposures were exposed to 30 puffs of air. All pump-puffing experiments were conducted within a P20 Purair (AirSience, Fort Myers, FL, United States) ductless fume hood equipped with a HEPA filter.

### Response Analysis and Pharyngeal Pumping Assay

Age-synchronized L4 larva (30–60) on NMG plates with food were exposed to 30 puffs of air, ECIG aerosol, and conventional cigarette smoke, as described above. Movement and responsiveness to plate vibration were assessed hourly for 12 h using an Olympus SZ51 Stereo Microscope. Three biological replicates were conducted for each condition.

Pharyngeal pumping assay was performed to investigate if the nematodes had undergone stress-induced sleep. Age-synchronized L4 larva (30–361) on NMG plates with food were exposed to 30 puffs of air, ECIG aerosol, and conventional cigarette smoke, as described above. Animals were assessed for pharyngeal pumping and counted hourly for 5 h followed by assessment at 10 h using an Olympus SZ51 Stereo Microscope. Pharyngeal pumping assessment involved observing individuals for 1–3 s intervals and the presence of pumping was counted if rhythmic opening and closing of the pharyngeal intestinal valve was readily apparent within the 3 s interval. Three biological replicates were conducted for each exposure condition. Pharyngeal pumping activity was expressed as percent animals pumping for each time point. All values are presented as the mean ± SEM.

### RNA Isolation and Quantitative RT-PCR

Age-synchronized L4 larva (∼50) on NMG plates with food were exposed to 30 puffs of air, ECIG aerosol and smoke, as described above. Total RNA was isolated 1, 5, and 24 h post exposure, as previously described (Hall et al., 2017). Briefly, animals were collected and incubated in K medium for 10 m to remove bacterial food from the intestinal lumen. *C. elegans* were collected by centrifugation (2000 rpm for 2 m) and rinsed once with K medium. The washed pellet was suspended in TRIZOL (Life Technologies Co., Grand Island, NY, United States) and transferred to tubes containing zirconia/silica beads. Nematode disruption was accomplished using a BeadBug Microtube Homogenizer (Benchmark Scientific Product, Edison, NJ, United States) with a 30 s agitation at maximum speed. RNA was extracted from the homogenate using phenol:cholorofom and isolated using Qiagen RNeasy kits (Qiagen Inc., Valencia, CA, United States), according to manufacturer’s instructions. The concentration of the purified RNA was assessed with a NanoDrop 8000 Spectrophotometer (Thermo Scientific^®^, Wilmington, DE, United States). For qRT-PCR, cDNA was generated from 55 ng of total RNA with RevertAid First Strand cDNA Synthesis Kit (Thermo Scientific^®^, Wilmington, DE, United States), according to manufacturer’s instructions. qRT-PCR was performed using QuantiTect SYBR Green RT-PCR kits (Qiagen) following manufacturer’s instructions in a QuantStudio3^®^ system (Applied Biosystems, Foster City, CA, United States). The primers used were: forward 5′-TGGATGTAAGGGAGACTGCAA-3′ and reverse 5′-CATTTTAATGAGCCGCAGCA-3′ for *mtl-1*; and forward 5′-AGTGTGACTGCAAAAACCAAAAT-3′ and reverse 5′-TAATGAGCAGCCTGAGCACAT-3′ for *mtl-2*. Each biological replicate was measured in triplicate and a minimum of three biological replicates were conducted for each condition.

To determine the induction levels of *mtl-1* and *mtl-2* to air, ECIG aerosol and smoke, *mtl-1* and *mtl-2* mRNA levels were normalized to *mlc-2* (myosin light chain). The primers used for *mlc-2* were: forward 5′-TTGACAGGAACTGACCCAGAGG-3′ and reverse 5′-ATAGCCTTGACCTCATCCTCG-3′. The log_2_ fold change in the steady-state *mtl-1* or *mtl-2* mRNA following exposure, compared to untreated (air) wild-type *C. elegans*, was then determined using the comparative *C*_T_ method (2^-ΔΔ^*^C^*_T_ method) (Schmittgen and Livak, 2008). All values are presented as the mean log_2_ fold change ± SEM.

### Statistical Analysis

Following each exposure treatment, the mean percentage (± SEM) of nematode pharyngeal pumping, as an index of stress-induced sleep, was recorded on an hourly basis for up to 5 h. Statistical differences in pharyngeal pumping between the treatment groups were determined using a two-way analysis of variance (ANOVA) and subsequent Bonferroni’s *post hoc* analysis. The mean log_2_ fold change (± SEM) for the 30 puffs air control group and all other treatment groups were recorded at 1, 5, and 24 h following treatment exposure and served as an index for *mtl-1* and *mtl-2* mRNA expression. Statistical differences in mRNA expression between the treatment groups were determined using a two-way ANOVA followed by Bonferroni’s *post hoc* analysis.

## Results

### Effects of Smoke and Aerosol on Initial Shock Response

Exposure to conventional cigarette smoke caused an initial shock response in the animals, followed by a delayed recovery period, whereas exposure to ECIG aerosol had little to no effect on movement and responsiveness (**Table 1**). None of the exposures caused lethality and all animals returned to normal movement behaviors by 9 h post exposure (**Table 1**). The most drastic reduction in movement was observed 1 h post exposure to conventional cigarette smoke. Movement behavior after this exposure appears to be concentration dependent and the animals displayed signs of movement and responsiveness similar to air control as early as 3 h after 15 puff, 5 h after 30 puff, and 9 h after 45 puff exposures (**Table 1**). In contrast, the initial response after exposure to ECIG aerosol showed movement and responsiveness similar to that of the air control and had little to no effect on movement and responsiveness for all puff amounts and time points (**Table 1**).

**Table 1:** Nematode movement response to exposure to air control, conventional cigarette smoke, and ECIG aerosol.

Treatment (puffs)		Hours post treatment					
		1	3	5	7	9	11
Air	30	++	++	++	++	++	++
Smoke	15	+	++	++	++	++	++
	30	+	+	++	++	++	++
	45	–	–	+	+	++	++
ECIG	15	++	++	++	++	++	++
	30	++	++	++	++	++	++
	45	++	++	++	++	++	++

*‘++’ indicates normal wild-type movement. ‘+’ indicates very little movement observed and/or movement only after stimulation. ‘–’ indicates no visible movement.*

### Effects of Smoke and Aerosol on Stress-Induced Sleep

**FIGURE 1:**
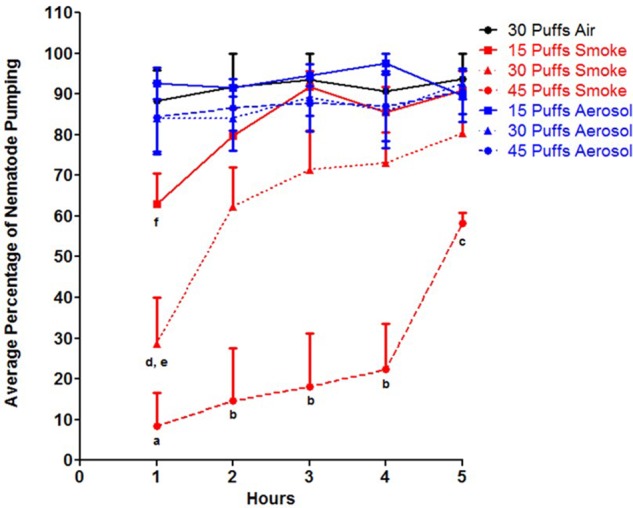
Percentage of animals that displayed pharyngeal pumping after exposure to conventional cigarette smoke (red lines), ECIG aerosol (blue lines) or air control (black lines) over time. Hours are post exposure. Means + SEM for three biological replicates are presented. As determined by two-way ANOVA: a = *p* < 0.001 for 45 puffs of smoke vs. control, 15 puffs of smoke, and all ECIG aerosol exposures at 1 h; b = *p* < 0.001 for 45 puffs of smoke vs. control, all smoke and all ECIG aerosol exposures at 2, 3, and 4 h; c = *p* < 0.05 for 45 puffs of smoke vs. control, 15 puffs of smoke, and all ECIG aerosol exposures at 5 h; d = *p* < 0.001 for 30 puffs of smoke vs. control and all ECIG aerosol exposures at 1 h; e = *p* < 0.01 for 30 puffs of smoke vs. control and 15 puffs of smoke at 1 h; f = *p* < 0.05 for 15 puffs of smoke vs. control and 15 puffs of ECIG aerosol at 1 h.

To determine whether the observed slow response phenotype after exposure to conventional cigarette smoke was due to stress-induced sleep, pharyngeal pumping was assessed. Pharyngeal pumping and locomotion are behavioral phenotypes observed while *C. elegans* sleep, and is noted as a key characteristic of stress-induced sleep (Trojanowski and Raizen, 2016; DeBardeleben et al., 2017). Exposure to conventional cigarette smoke resulted in significantly less percentage of individuals with pharyngeal pumping compared to both ECIG aerosol and air control as early as 1 h post exposure, with 45 puffs having the most drastic effect (**Figure 1**, *p* < 0.001). Additionally, the exposure to 45 puffs of conventional cigarette smoke was significantly different than 15 puffs at all time points tested and 30 puffs at time points after 2 h post exposure (**Figure 1**, *p* < 0.001). An initial percent pharyngeal pumping of 8.3 ± 8.3% was observed in response to conventional cigarette smoke along with a significantly delayed recovery response compared to the other exposures; an increase of only 14% by 4 h post exposure (**Figure 1**). Exposure to 30 puffs of conventional cigarette smoke resulted in a 28.5 ± 11.5% pumping at 1 h, significantly less than ECIG aerosol and air control as well as 15 puffs of conventional cigarette smoke (*p* < 0.001 and 0.01, respectively). Although at 1 h post exposure, 15 puffs resulted in 63.1 ± 7.3% pumping, which is approximately 7.5 times greater than the pumping activity 1 h post 45 puffs of conventional cigarette smoke, it was still significantly different than 15 puffs of ECIG aerosol and air control (**Figure 1**, *p* < 0.05). By 2 h post exposure, nematodes exposed to 30 puffs of conventional cigarette smoke restored pumping activity to 62.4 ± 9.7%, a level similar to 15 puffs of conventional cigarette smoke, ECIG aerosol, and air control. In contrast, animals exposed to 45 puffs of conventional cigarette smoke at 5 h post exposure was only 58.3 ± 2.6%, still significantly different than air control, all aerosol, and 15 puffs of conventional cigarette smoke (*p* < 0.05) (**Figure 1**).

By contrast, exposure to ECIG aerosol had a limited effect on pharyngeal pumping in the nematodes. All three exposure groups displayed similar amounts of pumping activity as compared to air control, in which pumping activity was above 80% in all ECIG exposure time points (**Figure 1**). Additionally, ECIG exposures were significantly different compared to conventional cigarette smoke at: 15 puffs, 1 h post (*p* < 0.05); 30 puff, 1 h post (*p* < 0.001); and 45 puffs, all time points (*p* < 0.05). Furthermore, all exposure groups (conventional cigarette smoke, ECIG, and air control), returned to 100% pumping activity 10 h post exposure (data not shown). Taken together, these data show that conventional cigarette smoke, but not ECIG aerosol, induces stress-induced sleep.

### Effects of Smoke and Aerosol on *mtl-1* and *mtl-2* Induction

**FIGURE 2:**
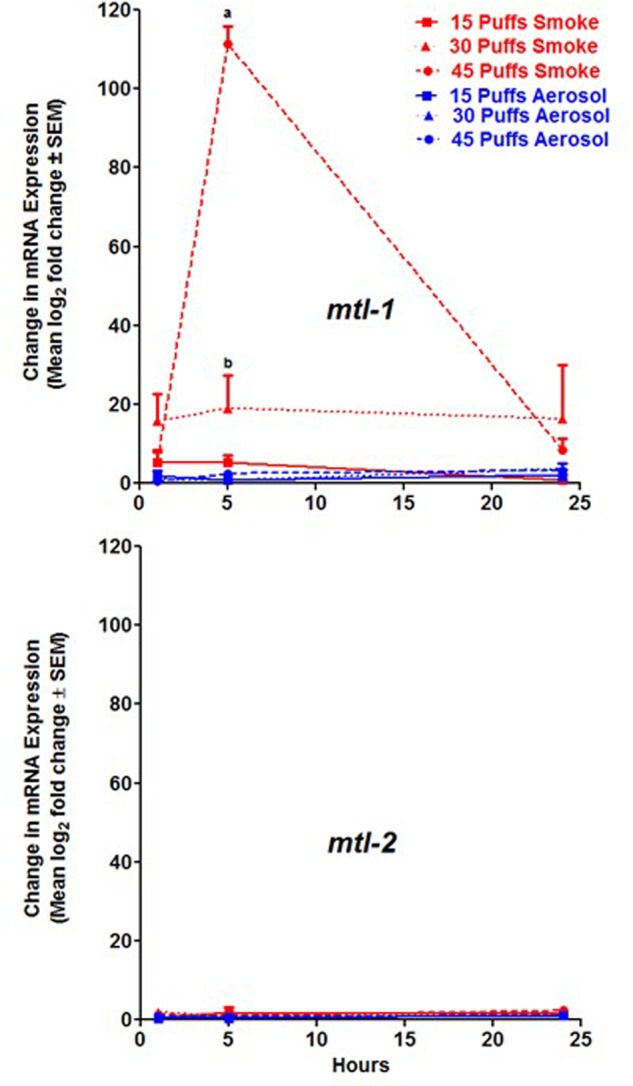
MT expression after exposure to conventional cigarette smoke (red lines) and ECIG aerosol (blue lines). *mtl-1* and *mtl-2* induction were measured 1, 5, and 24 h post exposure to 15, 30, and 45 puffs of treatment group. Means ± SEM for three biological replicates are presented. As determined by two-way ANOVA: a = *p* < 0.001 between 45 puffs smoke and all ECIG aerosol and 15 and 30 puffs smoke exposures at 5 h; and b = *p* < 0.05 between 30 puffs smoke and all ECIG aerosol exposures at 5 h.

The expression of *mtl-1* was greatest in *C. elegans* 5 h post exposure to 45 puffs of conventional cigarette smoke at a level of 111.4 ± 4.4 log_2_ fold change ± SEM compared to untreated and significantly different than all other conditions tested (**Figure 2**, *p* < 0.001). Initial increases in expression were observed 1 h post exposure for 15, 30, and 45 puffs of conventional cigarette smoke (**Figure 2**). Peak for all three exposures was 5 h with levels at 24 h resembling those of 1 h (**Figure 2**). ECIG aerosol exposure conditions resulted in little to no increase of *mtl-1* (**Figure 2**). In contrast, expression of *mtl-2* was not significantly increased in response to either conventional cigarette smoke or ECIG aerosol (**Figure 2**). Additionally, for both conventional cigarette smoke and ECIG, *mtl-2* expression levels were not significantly different when comparing hours post exposure (**Figure 2**). Thus, an induction in expression was only observed in *mtl-1*, and only in response to conventional cigarette smoke.

## Discussion

Based on the known harmful effects of conventional cigarettes, it was expected that a significant reduction of normal *C. elegans* outputs, as well as higher morbidity, would be observed after exposure to conventional cigarette smoke compared to ECIG aerosol. As expected, ECIG aerosol had little to no effect on *C. elegans*. However, *C. elegans* exposed to conventional cigarette smoke showed an initial decrease in movement and pharyngeal pumping, but recovered by 10 h after exposure for all puff exposures (**Figure 1** and **Table 1**). These data agree with a previous study that investigated *C. elegans* exposure to 4 h of continuous conventional cigarette smoke and found no effect on survival 24 h post exposure (Green et al., 2009). Additionally, a dose-dependent increase in the nicotine metabolite cotinine was found in the animals post exposure with levels returning to normal at 24 h, indicating that *C. elegans* are able to absorb material in conventional cigarette smoke through their cuticles (Green et al., 2009). The cuticle is similar in structure and function to the stratum corneum layer of human skin, serving as a protective barrier (Xu et al., 2012). Assuming these are analogous structures, toxins smaller than 500 KDa present in either ECIG aerosol or conventional cigarette smoke could readily diffuse through the cuticle (Bos and Meinardi, 2000). Therefore, the nematodes’ response to conventional cigarette smoke, impairment of locomotion, and pharyngeal pumping in this study suggests that toxins were absorbed in the *C. elegans*, resulting in stress-induced sleep, which was not observed in animals exposed to ECIG aerosol (**Figure 1**). This indicates that conventional cigarette smoke induces a much greater stress response in *C. elegans* compared to ECIGs.

Sleep is an evolutionarily conserved physiological response with implicated functions of energy conservation, macromolecular synthesis, memory, and clearance of metabolites from the brain. (Mignot, 2008; Xie et al., 2014; Gelaye et al., 2016). When *C. elegans* are exposed to high levels of environmental stressors, they become quiescent for a period of time before returning to normal. One hypothesis is that *C. elegans* utilize this mechanism to mitigate cellular stress and restore homeostasis. Induction of the stress-induced sleep phenotype in *C. elegans* includes heat, cold, hyperosmotic stress, ethanol, and tissue damage (Hill et al., 2014). Carbon monoxide is also known to induce a suspended animation, similar to stress-induced sleep, as a protection mechanism against hypoxia (Padilla et al., 2002; Nystul and Roth, 2004). Carbon monoxide is produced at a concentration of 831 ± 166 μM/L from smoke compared to a range between 0.006 ± 0.001 and 0.010 ± 0.003 μM/L from ECIG aerosol (Palazzolo et al., 2017a). *C. elegans* embryos were shown to undergo suspended animation in response to 24 h exposure of pure carbon monoxide with a recovery rate of 81.5% survival to adulthood (Nystul and Roth, 2004). This suggests that the observed stress-induced sleep at 45 puffs of conventional cigarette smoke may be a response to the carbon monoxide levels produced. Lastly, both hypoxic conditions and accumulation of ROS are known activators of epidermal growth factor signaling (EGF/EGFR) in humans (Tan et al., 2016). EGF/EGFR signaling in ALA neurons of *C. elegans* has proven to be essential for the induction of stress-induced sleep (Nath et al., 2016). Because sleep responses are well conserved across species, it is likely that the carbon monoxide conditions along with ROS may lead to the stress-induced sleep observed in this study in response to conventional cigarette smoke, but absent in the ECIG aerosol (**Figure 1**).

Interestingly, both conventional cigarette smoke and ECIG aerosol contain ROS-producing materials that can potentially induce stress-induced sleep in *C. elegans* (Williams et al., 2017; Palazzolo et al., 2017a), however, only conventional cigarette smoke led to this response (**Figure 1**). Evidence suggests that carbon monoxide in cigarette smoke induces hypoxia, which, in turn, triggers MT and Nrf-2 expression as a compensatory mechanism against oxidative stress (Zhou et al., 2017). On the other hand, 0.2% propylene glycol or a commercially available brand of E-liquid (V2 Platinum E-Liquid, V2CIGS/VMR Products LLC., Miami, FL, United States) containing ∼70% propylene glycol and either 0 or 2.4% nicotine (diluted to 0.14% propylene glycol and 48 ppm nicotine), in grape, menthol, or classic tobacco flavors, as well as distilled vapor extracts from E-liquid, have also been reported to induce a mild oxidative stress response in *C. elegans* through the Nrf-2 ortholog, SKN-1, after direct exposure to E-liquid (Panitz et al., 2015). Furthermore, other oxidative stress response genes such as the FOXO ortholog, DAF-16, did not elicit a response suggesting that SKN-1 plays a greater role in the detoxification/antioxidant response to E-liquid. It was shown that propylene glycol alone is sufficient to induce this oxidative stress in *C. elegans* (Panitz et al., 2015). Lastly, mild oxidative stress induced by ECIG-generated aerosol has been reported using *in vitro* cultures of a variety of human cell lines, but the oxidative stress induced by ECIG aerosol is generally far less than that produced by cigarette smoke (Anderson et al., 2016; Ji et al., 2016; Teasdale et al., 2016; Ganapathy et al., 2017).

The MTs are involved in ROS responses but play a more important role in the detoxification of heavy metals. In this present study, *mtl-1*, but not *mtl-2*, was transcriptionally activated in response to conventional cigarette smoke in a time- and dose-dependent manner (**Figure 2**). This concentration dependent expression of *mtl-1* is in line with the trends observed in the stress-induced sleep assay (**Figure 1**). Pharyngeal pumping activity in response to 15 and 30 puffs of conventional cigarette smoke had its highest increase in pharyngeal pumping recovery (16.7 and 33.9% increase, respectively) between 1 and 2 h post exposure, which correlates with *mtl-1* expression resulting in little to no increase in expression between the 1 and 5 h time points (**Figure 2**). In contrast, exposure to 45 puffs of smoke resulted in a delayed recovery period between 4 and 5 h post exposure and *mtl-1* expression peaking at 5 h (111.4 ± 4.4 log_2_ fold change ± SEM). This relationship was not observed in nematodes exposed to ECIG aerosol.

Both MTL-1 and MTL-2 function in detoxification but they are structurally different and have been found to respond differently to various toxicants and stressors (Freedman et al., 1993; You et al., 1999; Zeitoun-Ghandour et al., 2010). Specifically, MTL-2 has been shown to have a higher affinity for Cd (Zeitoun-Ghandour et al., 2010). Considering the lack of expression of *mtl-2* in this study in response to conventional cigarette smoke (**Figure 2**), it can be suggested that Cd is likely not the only contributor to the toxicity of cigarettes. Trace amounts of Cd were found in conventional cigarette smoke (0.062 ± 0.008 μg) as well as ECIG aerosol (0.047 ± 0.003 μg) along with other metals (Al, Cu, Fe, Mn, Pb, and Zn) and As, with concentrations in ECIG aerosol at lower or comparable levels to that found in conventional cigarette smoke (Palazzolo et al., 2017a). These levels are thought to be the result of metals leaching from the ECIG device (Palazzolo et al., 2017a). The lack of observed *mtl-2* expression (**Figure 2**) is consistent with previous studies in which it was not significantly increased in response to Cu, Zn, Ni, Pb, and As (Ma et al., 2009; Anbalagan et al., 2012). Additionally, work investigating metals in soils suggests that *mtl-2* expression is reduced after exposure to a combination of heavy metals (Anbalagan et al., 2012).

The MTs are highly conserved from nematodes to humans. Four isoforms (MT-1-4) have been characterized in mammals and their expression is tissue specific, with MT-1 and MT-2 being ubiquitously expressed in all tissues (Haq et al., 2003). Studies have shown that MT-1 and MT-2 are induced by a variety of metals including Zn, Cd, Cu, and to some degree Ni, all of which are components found in both conventional cigarette smoke and ECIG aerosol (Palmiter, 1994; Mikheev et al., 2016; Palazzolo et al., 2017a). Additionally, inorganic As and Cd alone as well as in combination with contaminated water samples were shown to increase expression of various MT-1 isoforms in placental cells, peaking at 4 h post treatment of Cd plus the contaminated water sample and 8 h post treatment of inorganic As plus the contaminated water (Adebambo et al., 2015). This time frame of response correlates with the peak of *mtl-1* gene expression observed in this study in response to 45 puffs of conventional cigarette smoke (**Figure 2**), suggesting a conserved response between *C. elegans mtl-1* and mammalian MT-1.

From a physiological perspective, we are confident that cigarette smoke has a more dramatic effect compared to ECIG-generated aerosol on stress-induced sleep, an initial stress response, and the induction of *mtl-1*. However, this investigation is not without its limitations. First, the outcomes of this study, especially *mtl-1* and *mtl-2* expression, were determined using the nematode, *C. elegans*, and not mammalian tissue. Even so, the MT genes are highly conserved in both structure and function. Due to a lack of a respiratory system in *C. elegans, in vitro* studies of MT expression in mammalian or human respiratory cells would better establish whether or not the trace metals in ECIG aerosol are a health risk. Despite being simplistic in nature, *C. elegans* does offer the ability to look at stress responses in a live intact organism, as compared to *in vitro* cell culture studies. It is also important to remember that many of the health effects observed from cigarettes are caused by chronic exposure to conventional cigarette smoke, whereas this study compared acute responses to ECIG aerosol and conventional cigarette exposures. Chronic exposure or generational effects could be tested using the nematodes to assess points of interest in relationship to possible effects. Another limitation is that this study utilized only one rendition of E-liquid (i.e., 50% propylene glycol and 50% glycerol, containing 20 mg/ml of nicotine and no flavors). It is entirely possible that other variations of E-liquids, particularly those containing additional flavorings, could induce more severe outcomes in *C. elegans*, as shown by others in several human cell lines (Bahl et al., 2012; Leigh et al., 2016; Sundar et al., 2016). It has been shown that when exposing the nematodes directly to E-liquid, nicotine, regardless of solvent, played a role in body size and reproduction (Panitz et al., 2015). More surprisingly, the propylene glycol, regardless of nicotine content, had the greatest effect and only the classic tobacco additive resulted in any changes of the overall effect to E-liquid (Panitz et al., 2015). However, Panitz et al. (2015) looked at directly exposing the nematodes to the E-liquid, whereas this present study exposed the animals to aerosolized E-liquid, thus a difference in the delivery method and ultimately the exposure route, might lead to differences in the effects of the various chemicals. Finally, while the presence of a number of trace metals and carbon monoxide, in both ECIG aerosol and conventional cigarette smoke, have been previously quantified by our laboratory (Palazzolo et al., 2017a), we can only speculate that these substance are indeed absorbed by *C. elegans* and are biologically active to induce the observations reported in this investigation. Consequently, measuring the amount of trace metals absorbed by *C. elegans* exposed to ECIG aerosol and conventional cigarette smoke, using inductively couple plasma and mass spectrometry, is a logical next step. Furthermore, it should also be mentioned that cigarette smoke contains thousands of other compounds of which many, either alone or in combination, could possibly induce *mtl-1* or *mtl-2* expression if absorbed by the nematodes. Of course, there is no way of positively knowing which of these other compounds in conventional cigarette smoke could also affect MT expression or if any of these compounds have competing effects on them without testing each known compound individually. Likewise, this also holds true for ECIG aerosol even though it consists of considerably fewer compounds. The possibility exists that the effect of one compound in the aerosol may induce *mtl-1* and/or *mtl-2* while another compound may suppress *mtl-1* and/or *mtl-2* thus making it appear that ECIG aerosol has no effect on MT expression, when in fact different compounds of the ECIG aerosol could have antagonistic effects.

This study aimed to assess the relative safety of ECIG aerosol by comparing MT expression and physiological response outputs in *C. elegans*. The data demonstrate that ECIGs do not induce a stress response and that no MT expression was found, suggesting little to no ROS present after exposure. Further investigation of the toxicological effects of trace metals, and other constituents of aerosolized E-liquid, is needed before establishing ECIGs as a safe alternative to conventional cigarettes. MT expression after exposure to comparable levels of trace metals (Al, Cu, Fe, Mn, Pb, and Zn) and As found in both conventional cigarette smoke and ECIG aerosol, individually and in combination, can tease out which components might contribute to the response and more specifically, the ROS responses. Another concern is the long-term effects of ECIG exposure. Chronic exposure of *C. elegans* to ECIG aerosol over time could help to shed light on these effects. These data, along with previous studies, suggest that ECIGs, although not completely harmless, may be a safer alternative to conventional cigarettes.

## Author Contributions

EC conducted the experiments. JH oversaw the experiments and conducted the data analysis. DP provided insight into the project. EC, JH, and DP contributed to the writing of the manuscript.

## Conflict of Interest Statement

The authors declare that the research was conducted in the absence of any commercial or financial relationships that could be construed as a potential conflict of interest.
